# Lactose Dehydrogenase in Patients with Severe COVID-19: A Meta-Analysis of Retrospective Study

**DOI:** 10.1017/S1049023X20000576

**Published:** 2020-04-24

**Authors:** Xiaoyi Huang, Fengxiang Wei, Ziqing Yang, Min Li, Liuhong Liu, Ken Chen

**Affiliations:** 1. School of Nursing, Guangdong Pharmaceutical University, Haizhu District, Guangzhou, China; 2. Shenzhen Longgang District Maternity & Child Healthcare Hospital of Shenzhen City, Shenzhen, China

**Keywords:** coronavirus, COVID-19, lactose dehydrogenase

To the Editor:

Coronavirus disease 2019 (COVID-19) is another human infectious disease caused by coronavirus.^1,2^ COVID-19 is a new infectious disease in the world whose origin, pathological mechanism, and treatment strategy are not fully understood. In the course of disease development and treatment, laboratory examination is very important to monitor the change of the disease and evaluate the therapeutic effect.^3^ As of March 11, 2020, the World Health Organization (WHO; Geneva, Switzerland) declared the COVID-19 outbreak a global pandemic.^4,5^ COVID-19 may become a globally heavy burden, especially in the low-income and middle-income countries.^6^ The diagnosis, changes, and treatment evaluation of the disease need to be tested to provide an essential basis. During the progress of the disease, multiple organs such as lung, kidney, liver, and heart will be damaged, and whether these organs are destroyed or not, and the degree of damage, depends on the results of clinical tests.^7^ We performed an analysis of the current scientific study to investigate whether lactose dehydrogenase (LAC) may play a role in distinguishing the presence of severe COVID-19 in patients with viral infection, and whether its value changed substantially in patients with viral infection.

A systematic review was conducted in accordance with the Preferred Reporting Items for Systematic Reviews and Meta-Analyses (PRISMA) guidelines. A systematic search of articles published from January 1989 to March 11, 2020 was carried out using PubMed (National Center for Biotechnology Information, National Institutes of Health; Bethesda, Maryland USA), Embase (Elsevier; Amsterdam, Netherlands), Cochrane Library (The Cochrane Collaboration; London, United Kingdom), Web of Science (Thomson Reuters; New York, New York USA), CNKI (Tsinghua University; Beijing, China), and other databases. The databases were searched using the terms “Clinical Features” AND “COVID-19” OR “2019-nCoV.” The literature quality was evaluated and the data were extracted by two researchers, and statistical analysis was performed using Review Manage 5.3 software (Cochrane; London, United Kingdom). The pre-specified criteria were used by two reviewers to screen the titles, abstracts, and full texts of the relevant articles. A meta-analysis was then performed for calculating the individual and pooled odds ratios (OR); their relative 95% confidence interval (CIs) were calculated by random effects models and fixed-effect models. Heterogeneity between studies was evaluated by analyzing the characteristics of the studies and quantitatively assessed using the *I* ^2^ statistic. A *P* value equal to or less than .05 was considered as the statistically significant level.^8^

**Figure 1.**
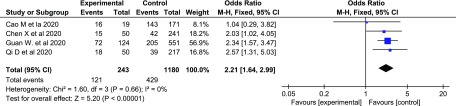
The Forest Figure of LAC Values in COVID-19 Patients with or without Severe Disease.

Abbreviations: COVID-19, coronavirus disease 2019; LAC, lactose dehydrogenase.

In total, 68 articles could initially be identified using these search criteria. A total of 20 articles were excluded because they were duplicates, 28 articles were excluded after the title and abstract screening, and 16 articles were excluded after full-text screening. Finally, four articles complied with the inclusion criteria. Overall, this meta-analysis ended up incorporating four studies. Retrospective analysis was performed on 1,855 COVID-19 cases, and 292 cases presented severe COVID-19. Depending on this study, the overall morbidity rate of COVID-19 is 15.7%. Here, age of COVID-19 patients is predominantly concentrated to 46-50 years old (Table 1 ^1,9–11^). The research results show that compared with the conventional treatment group, LAC increased protectionism values are associated with a two-fold higher risk of severe COVID-19 (OR = 2.21; 95% CI, 1.64-2.99; Figure 1).

**Table 1.** Characteristics of Studies Included in the Meta-Analysis

Author	Study Location	Average Age	N. Cases	Setting	Sex (NO.)	Research Type
Cao M^9^ et al 2020	Shanghai,China	50	198;19 severe	Hospitalizedpatients	Male: 101Female: 97	Retrospective Study
Chen N^1^ et al 2020	Hunan,China	46	291;50 severe	Hospitalizedpatients	Male: 145Female: 146	Retrospective Study
Guan W^10^ et al 2020	30 provinces,China	47	1099;173 severe	Hospitalizedpatients	Male: 640Female: 459	Retrospective Study
Qi D^11^ et al 2020	Chongqing,China	48	267;50 severe	Hospitalizedpatients	Male: 149Female: 118	Retrospective Study

Although the increase in LAC in COVID-19 patients appears to be limited, as highlighted in a recent article, the results of this concise meta-analysis will indicate that continuous LAC measurements may play a role in predicting the evolution of more severe disease forms. The evidence makes a plausible explanation. The increase of LAC in severe patients is more obvious than that in normal patients, which may be linked to the inflammatory response. The sharp and sudden rise of LAC accompanied by respiratory failure often indicates the acute inflammatory response storm and indicates the disease progression.

The LAC test is not widely used in clinical tests. The LAC in the body mainly comes from the results of anaerobic fermentation of glucose by red blood cells and tissue cells. Glucose is paralyzed by a series of enzymes to form pyruvate, which is then catalyzed by LAC to form LAC, which is circulated through the blood to the liver and converted into glucose via gluconeogenesis. Severe hypoxia, strenuous exercise, and severe liver disease all lead to the increase of LAC level.^12^ Changes in LAC levels in COVID-19 patients can reflect the body’s hypoxia state. In hospitals where blood and gas analysis cannot be conducted, LAC measurement can indirectly reflect the body’s respiratory function and also reflect the degree of metabolic acidosis in the body.^13^

Patients with COVID-19 showed mild or normal symptoms after onset and were discharged after treatment.^14^ However, with the development of the disease, some patients become severe or critical, with dyspnea and hypoxemia, and progress to acute respiratory distress syndrome (ARDS) and severe metabolic acidosis, which can lead to death.^15^ Due to the complexity of severe patients and emerging infectious diseases, it is inevitable that there are errors or deficiencies which require more and more in-depth research in many aspects, as well as the joint efforts of multiple disciplines such as epidemiological investigation, clinical medicine, pathology, laboratory medicine, and pharmacology.^16^
